# Efficacy and safety of intermittent theta-burst stimulation in patients with schizophrenia: A meta-analysis of randomized sham-controlled trials

**DOI:** 10.3389/fphar.2022.944437

**Published:** 2022-08-22

**Authors:** Kah Kheng Goh, Chun-Hsin Chen, Tzu-Hua Wu, Yi-Hang Chiu, Mong-Liang Lu

**Affiliations:** ^1^ Department of Psychiatry, Wan Fang Hospital, Taipei Medical University, Taipei, Taiwan; ^2^ Psychiatric Research Centre, Wan Fang Hospital, Taipei Medical University, Taipei, Taiwan; ^3^ Department of Psychiatry, School of Medicine, College of Medicine, Taipei Medical University, Taipei, Taiwan; ^4^ Department of Clinical Pharmacy, School of Pharmacy, College of Pharmacy, Taipei Medical University, Taipei, Taiwan.

**Keywords:** schizophrenia, theta-burst stimulation, meta-analysis, efficacy, safety

## Abstract

Theta-burst stimulation is a non-invasive brain stimulation technique that was introduced as a potential augmentation treatment for patients with schizophrenia. The purpose of this meta-analysis was to investigate the therapeutic efficacy and safety of intermittent theta-burst stimulation in patients with schizophrenia. Following the PRISMA guidelines, the MEDLINE, Embase, Cochrane, Scopus, Web of Science, and CNKI databases were searched for relevant studies from database inception to 9 January 2022. Change in symptom severity among patients with schizophrenia was the primary outcome, and changes in cognitive function and safety profiles, including the discontinuation rate and adverse events, were secondary outcomes. In total, 13 double-blind randomized sham-controlled trials with 524 patients were included. Intermittent theta-burst stimulation adjunct to antipsychotics was associated with significantly improved psychopathology in patients with schizophrenia, particularly for negative symptoms and general psychopathology but not for positive symptoms or cognitive function. The stimulation parameters influenced the effectiveness of intermittent theta-burst stimulation. A more favorable effect was observed in patients who received theta-burst stimulation at the left dorsolateral prefrontal cortex, with ≥1800 pulses per day, for ≥20 sessions, and using an inactive sham coil as a placebo comparison in the study. The intermittent theta-burst stimulation is well tolerated and safe in patients with schizophrenia. Intermittent theta-burst stimulation adjunct to antipsychotics treatment is associated with significant improvement in negative symptoms and favorable tolerability in patients with schizophrenia. This meta-analysis may provide insights into the use of intermittent theta-burst stimulation as an additional treatment to alleviate the negative symptoms of schizophrenia.

## 1 Introduction

Schizophrenia is a chronic and disabling mental disorder that affects 0.3%–0.7% of the general population worldwide (McGrath et al., 2008). It is characterized by two primary symptom domains: positive symptoms (delusions, hallucinations, and disorganized thoughts) and negative symptoms (blunted affect, alogia, anhedonia, asociality, and avolition). Other forms of common psychopathologies, for example, anxiety, depression, active social avoidance, uncooperativeness, poor attention, and poor impulse control, are summarized by one general underlying psychopathology of schizophrenia. Furthermore, there is growing awareness that cognitive symptoms, for instance, impaired memory, concentration, and executive functioning, need to be considered to gain a thorough understanding of the etiology and for better function restoration in schizophrenia (Schaefer et al., 2013). Although antipsychotics are the treatment of choice for schizophrenia, approximately one-third of patients exhibit a poor response (Elkis and Buckley, 2016). In patients with schizophrenia who are unresponsive to antipsychotics, nonpharmacological treatment strategies may be tailored to improve their symptoms (Kane et al., 2019).

Electroconvulsive therapy (ECT) is indicated for patients with schizophrenia who are treatment-resistant, but it has been limited its use to its need for anesthesia and its cognitive side effects (Grover et al., 2019). Increasing attention has been drawn to the implication of newly invented brain stimulation strategies for schizophrenia. Repetitive transcranial magnetic stimulation (rTMS) is a non-invasive technique that can modulate the activity of targeted cortical areas and their associated networks as well as spreading across networks (Beynel et al., 2020). Randomized sham-controlled trials of rTMS in patients with schizophrenia have yielded inconsistent results due to differences in the assessment tools, patient characteristics (e.g., baseline psychopathology and duration of illness), and rTMS protocol parameters (e.g., stimulation frequency, stimulation strength, targeted brain area, total stimulation sessions, and number of stimulation pulses per session) (He et al., 2017). However, accumulating evidence indicates that rTMS is a potential strategy for ameliorating both positive and negative symptoms in patients with schizophrenia, but not cognitive symptoms (Osoegawa et al., 2018; Li J et al., 2020; Guttesen et al., 2021; Sciortino et al., 2021).

Theta-burst stimulation (TBS), a variation of rTMS, consists of three bursts of 50-Hz stimulation with a 200-ms interval between bursts at an active motor threshold intensity of 80%–120%, corresponding to theta brain oscillations (Huang et al., 2005; Blumberger et al., 2018). Compared with rTMS, TBS requires a shorter stimulation duration and lower stimulation pulse intensity, and it exerts more impact on synaptic plasticity (Rounis and Huang, 2020; Ferro et al., 2022). Theoretically, TBS transiently alters cortical excitability in the brain circuits through the accumulation of glutamate and gamma-aminobutyric acid, based on temporal pattern and level of the trigger factor, as well as NMDA-receptors that involved (Huang et al., 2005; Huang et al., 2007). The direction and amount of the after-effect are determined by the sum of excitatory or inhibitory substances that are critical in determining whether a synapse undergoes long-term potentiation or long-term depression. Excitatory after-effects are more rapid and shorter-lasting while inhibitory after-effects take longer to cumulate (Rounis and Huang, 2020). Two stimulation patterns of TBS, continuous theta-burst stimulation (cTBS) and intermittent theta-burst stimulation (iTBS), are resulted in different after-effects. In contrast to cTBS which leads to long-term depression-like reduction of cortical excitability after providing a 40 s continuous stimulation consisting of 600 pulses, iTBS involves 600 pulses that are delivered in a 2 s strains that are repeated every 10 s for 20 cycles and leading to long-term potentiation-like effects of cortical excitability (Huang et al., 2005). The therapeutic efficacy of iTBS has been proven and its protocol was cleared by the Food and Drug Administration (FDA) as a treatment option for major depressive disorder in August 2018. Albeit the FDA approvals, the mechanisms underlying excitability changes and the dose-dependency of iTBS remain poorly understood. Different parameter space of iTBS, including location of stimulation target, focality of stimulation and depth of target, frequency of stimulation, pulse intensity, and duration of stimulation are accounted for the clinical effects (Hurtado-Puerto et al., 2020).

Given the role of iTBS in clinical treatment of mental disorders, various studies have examined the potential clinical benefits of iTBS in patients with schizophrenia. Despite the increased interest in this area, consensus regarding the efficacy of iTBS augmentation for the treatment of schizophrenia is lacking. Several randomized controlled trials (RCTs) of iTBS in patients with schizophrenia have been published, which encouraged us to investigate its effectiveness and safety. Therefore, we conducted this meta-analysis to provide an update on the therapeutic effect and safety of iTBS in patients with schizophrenia, and we further investigated the effects of potential influencing factors, including the parameter space of iTBS.

## 2 Materials and methods

The meta-analysis protocol is registered at PROSPERO (http://www.crd.york.ac.uk/PROSPERO, registration number CRD42021265299) and was in accordance with the Preferred Reporting Items for Systematic Reviews and Meta-analyses (PRISMA) guidelines (Moher et al., 2009).

### 2.1 Selection criteria

Selection criteria for studies were structured in accordance with the Participants, Interventions, Comparisons, Outcomes reporting structure.

#### 2.1.1 Participants

Patients with schizophrenia, who were diagnosed based on any recognized diagnostic criteria, including the Diagnostic and Statistical Manual of Mental Disorders, International Statistical Classification of Diseases and Related Health Problem, and Chinese Classification of Mental Disorders, were included.

#### 2.1.2 Interventions

All double-blind, randomized, sham-controlled trials of iTBS in patients with schizophrenia were included. To compare the effects of different stimulation parameters (i.e., brain target, navigation method, coil type, and stimulation sessions), no restriction was applied to the iTBS treatment protocol.

#### 2.1.3 Comparisons

Sham groups were defined as those who were treated with sham control (i.e., coil angled on the scalp or use of a sham coil). Studies with a comparison of other treatment modalities were excluded.

#### 2.1.4 Outcomes

Change in symptom severity among patients with schizophrenia was the primary outcome. Change in psychopathology was measured using differences in Positive and Negative Syndrome Scale (PANSS) scores between baseline and study endpoint. The PANSS is used for measuring the symptom severity of positive symptoms, negative symptoms, and general psychopathology symptoms among patients with schizophrenia (Kay et al., 1987). Cognitive function improvement and safety profile (e.g., discontinuation and adverse-event rates) were defined as the secondary outcomes.

No restrictions were imposed on the type of iTBS protocols used in eligible studies. Similarly, no restrictions were applied on the type of treatment prior to inclusion; the duration of treatment; whether administration was adjunctive or monotherapy; where the study was conducted; or the age, sex, and ethnicity of the study participants. However, only studies with adult participants were included. Studies that employed both parallel and crossover designs were included; however, for crossover studies, to avoid carryover effects and possible loss of blinding integrity, only the results from the initial randomization were included (Krogh et al., 2019). Studies that started concomitantly with new antipsychotics were excluded in order to measure the effectiveness on the psychopathology was mainly brought by the introduction of iTBS. Non-RCT studies, such as observational studies, case reports, reviews, commentaries, conference proceedings, and open-label studies, were excluded. No publication language restriction was applied, but the availability of an English abstract was required.

### 2.2 Search strategy

Two independent authors (KKG and MLL) designed the search strategy with the following search terms: “theta burst stimulation OR TBS OR theta burst transcranial magnetic stimulation OR transcranial theta burst stimulation” AND “schizophrenia OR schizo* OR psychotic OR psychosis”. The MEDLINE, Embase, Cochrane, Scopus, Web of Science, and CNKI databases were searched from database inception to 09 January 2022. The protocols of eligible studies were obtained from ClinicalTrials.gov (https://clinicaltrials.gov/) or other clinical trials registry platforms to confirm that all included studies met the aforementioned inclusion and exclusion criteria. All duplicates were excluded. The titles and abstracts of the articles were screened for adherence to the inclusion and exclusion criteria. The references listed in selected articles were checked thoroughly to screen for additional articles that could be included.

### 2.3 Data extraction

Data for all studies were extracted independently by two researchers (KKG and MLL). The following details were abstracted for each included study: (a) study characteristics (primary author, publication year, country, context in which the study was conducted, duration of the intervention, study design, case number, sex ratio, and final study results); (b) characteristics of the study population (age, sex, antipsychotic dosage, duration of illness, and baseline psychopathology severity); (c) details of the intervention (stimulation protocol, targeted brain area, navigation method, coil type, stimulation session, stimulation strength, and type of sham control); (d) primary outcome (change in psychopathology); and (e) secondary outcomes (change in cognitive function, discontinuation rate, and adverse-event rate). Indirect measurements were made according to the principles and guidelines provided in the Cochrane Handbook for Systematic Reviews of Interventions Version 5.1.0 (Higgins et al., 2011). Corresponding authors of eligible studies were contacted by email in the event of incomplete or partly unavailable results. All available data were sought from authors until the date before the final analysis.

### 2.4 Quality assessment

The risk of bias in the included studies was assessed using the Cochrane Risk of Bias Assessment Tool version 2, for both cluster-randomized trials and crossover trials (Sterne et al., 2019). Two authors (KKG and MLL) independently assigned a high or low rating to all bias domains, namely randomization process, deviations from the intended interventions, missing outcome data, measurement of the outcome, and selection of the reported result. Any discrepancies between the two authors were resolved through consensus. The quality of evidence for the primary and secondary outcomes was evaluated using GRADE criteria (Guyatt et al., 2008).

### 2.5 Data synthesis and statistical analysis

The characteristics of the included studies, including the primary author, publication year, country, study population and context, study duration, intervention variables, use of adjunctive antipsychotics, patient age, patient sex, duration of illness, baseline severity, and iTBS effectiveness, are summarized. The intention-to-treat principle was applied for subsequent analysis.

Therapeutic effects of iTBS are summarized by calculating standardized mean differences (SMD) for change in symptom severity in patients with schizophrenia, and discontinuation and adverse-event rates were determined by calculating the risk ratios (RR) of the included studies. Random-effects models were applied to assess the heterogeneity among the included studies, assuming that the effects estimated in the included studies were not identical but followed certain distributions. For continuous outcomes, DerSimonian and Laird random-effects models with inverse-variance weighting were used to summarize the effects across studies and estimate the SMDs and their corresponding 95% confidence intervals (CIs). For binary outcomes, Mantel–Haenszel random-effects models were used to analyze the pooled RRs and 95% CIs. Two-sided *p* values were calculated for each outcome. Pooled results were only considered when at least two adequately powered studies were available to avoid underpowered results (Turner et al., 2013). To minimize the disparity in measurements, the total Scale for the Assessment of Negative Symptoms (SANS) scores were converted to PANSS negative subscale scores using the following simple equation obtained through regression analysis as PANSS negative score = 7.1196 + (0.3362 × total SANS scores) while total Scale for the Assessment of Positive Symptoms (SAPS) scores were converted to PANSS positive subscale scores as PANSS positive score = 11.1886 + (0.2587 × total SAPS scores) (van Erp et al., 2014). The meta-analysis was performed using Review Manager (RevMan) version 5.4.0 for Mac OS (The Nordic Cochrane Centre, The Cochrane Collaboration, Copenhagen, Denmark).

Heterogeneity among the studies was quantified using the χ^2^ test and the I^2^ statistic, with *p* < 0.05 and I^2^ > 50% indicating moderate heterogeneity. Exploratory meta-regression was applied to explore the heterogeneity among baseline characteristics (i.e., age, gender, severity of baseline psychopathology, duration of illness, antipsychotic dosage, and overall study duration) and stimulation parameters. Further, a stratified meta-analysis was conducted with an *a priori* subgroup to explore heterogeneity in the estimated effects between different populations and to identify the potential moderators or mediators of the reported outcomes, especially for different protocols and stimulation parameters of iTBS (i.e., targeted brain area, number of stimulation sessions, number of stimulation pulses, stimulation strength, and type of sham control), and the subgrouping was defined according to the median of the parameters.

To examine the robustness of the study outcomes, we conducted sensitivity analyses for study quality, alternative statistical approach (fixed-effects models), study design, population, sample size, publication language, and diagnostic criteria used in the study. Publication bias was evaluated using funnel-plot asymmetry, Begg and Mazumdar rank correlation, Egger’s regression, the Fail-safe N test, and Duval and Tweedie’s trim and fill method. Egger’s regression was used to test the asymmetry of the funnel plot, with *p* < 0.05 indicating publication bias. If the results for publication bias obtained from the above analyses were conflicting, then Duval and Tweedie’s trim and fill method was applied; this method assumes that the most extreme results were not published, and the effect size is re-estimated by imputing these missing studies. A smaller change in the effect size during adjustment with the trim and fill method indicates a higher accuracy in the initial effect size (Idris, 2012). Meta-regression and publication bias were analyzed using Comprehensive Meta-Analysis Version 3 (Biostat, Englewood, NJ, United States).

## 3 Results

### 3.1 Identification of eligible studies

**FIGURE 1:**
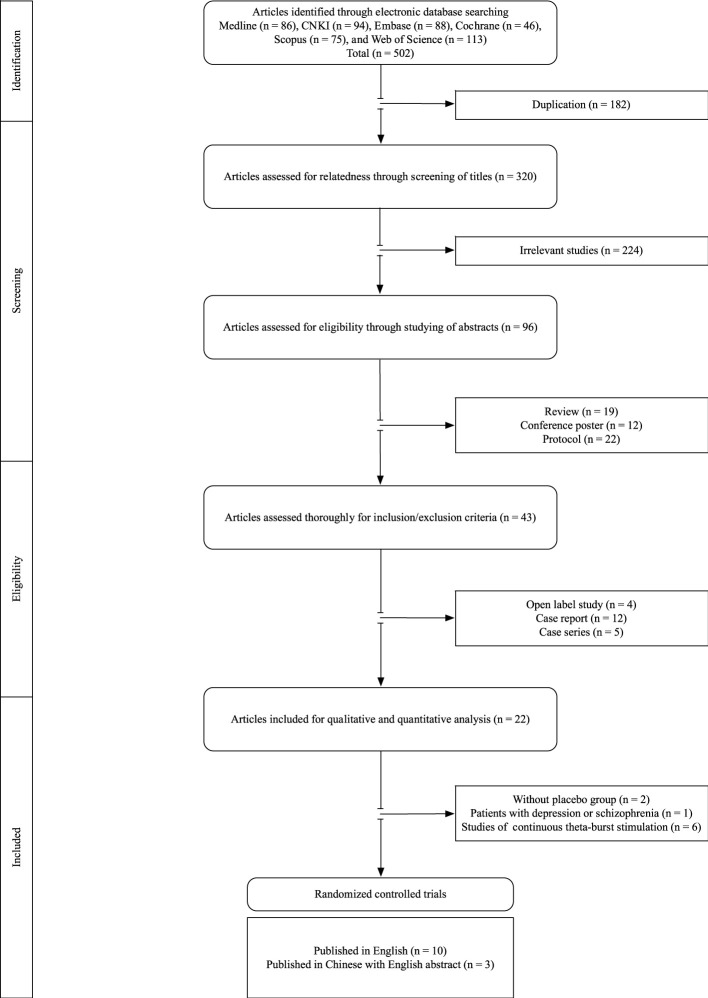
PRISMA flowchart of the study selection.

A total of 502 articles were retrieved, and after the removal of irrelevant studies and duplicates, 96 remained. The study selection process is shown in Figure 1. Overall, 43 articles were assessed for relatedness and eligibility by studying their full-text versions to determine whether they met our inclusion and exclusion criteria; 21 studies were found ineligible. Of the remaining articles, six were excluded for using cTBS protocols, two were excluded for the lack of a sham group for comparison (Kindler et al., 2013), and one study was excluded due to its inclusion of both patients with schizophrenia and patients with depression (Bodén et al., 2021), leaving 13 for qualitative and quantitative analyses.

### 3.2 Study and patient characteristics

This meta-analysis included 13 studies in which iTBS was used to treat patients with schizophrenia (Chen et al., 2011; Kazemi et al., 2012; Zheng et al., 2012; Zhao et al., 2014; Zhen et al., 2015; Zhu et al., 2019; Walther et al., 2020; Wang et al., 2020; Basavaraju et al., 2021; Bation et al., 2021; Chauhan et al., 2021; Wu et al., 2021; Zhu et al., 2021). The included studies were published between 2011 and 2021. Table 1 summarizes the characteristics of the included studies; including one study that compared different protocols of iTBS using a three-arm sham-controlled crossover design (i.e., cTBS, iTBS, and sham control) (Walther et al., 2020). With regard to most studies that delivered more than 10 sessions, one study that only administered one session of iTBS (Walther et al., 2020) was excluded for efficacy analysis. iTBS was delivered adjunct to antipsychotics in patients with schizophrenia in all the included studies. Of these studies, 10 were published in English, and three were published in Chinese with an English abstract (Zheng et al., 2012; Zhen et al., 2015; Zhu et al., 2019).

**TABLE 1:** General characteristics of randomized controlled trials of intermittent theta-burst stimulation in patients with schizophrenia.

Study; Country	Population; Severity; Setting	Coil; Navigation method	Duration of Tx/End^c^	Daily sessions; Total sessions; PPS; PPD; Total pulse delivered	rMT (%)	Treatment protocol	*n*	Male	CPZEq (mg/d)		Mean age (year)		Illness duration (year)		b-PANSS scores		Efficacy^e^		
									*M*	*SD*	*M*	*SD*	*M*	*SD*	*M*	*SD*	T	P	N
Brain target: Left dorsolateral prefrontal cortex																			
Bation et al. (2021) France	Sch (DSM-IV); Neg; TRS; b-SANS ≥20; …	Figure-8; 6 cm^a^	10d/6m	2; 20; 990; 1980; 19800	80	iTBS	12	1.00	325	206	42.3	9.4	15.0	5.9	78.83	7.96	✗	✗	✓
						Inactive Sham	10	0.90	389	171	41.6	12.6	17.1	15.4	71.50	14.58			
Chen et al. (2011) China	Sch (DSM-IV); Neg; b-PANSS-N ≥20; IPD	Circular; F3	20d/4w	4; 80; 600; 2400; 48000	80	iTBS	23	0.70	505	153	37.4	11.8	17.0	24.4	74.61	5.84	✓	✗	✓
						Inactive Sham	19	0.58	466	131	39.7	13.3	13.0	22.2	74.95	6.56			
Kazemi et al. (2012) Iran	Sch (DSM-IV); b-PANSS-P/N ≥15; …	Figure-8; 5 cm^a^	20d/4w	1; 20; 600; 600; 12000	80	iTBS	5	…	…	…	26.6	4.7	3.6	1.9	113.60	15.57	✗	✗	✓
						Inactive Sham	5	…	…	…	27.6	2.5	4.6	2.1	115.60	18.42			
Wang et al. (2020) China	Sch (DSM-IV); b-ΔPANSS <10%; OPD	Figure-8; MRI-Nv	14d/2m	3; 42; 600; 1800; 25200	80	iTBS	25	0.44	488	338	24.0	4.4	5.1	3.8	63.88	14.49	✓	✗	✓
						Inactive Sham	25	0.44	350	292	26.6	9.0	4.9	5.3	63.91	16.31			
Wu et al. (2021) China	Sch (DSM-IV);…; OPD	Figure-8; MRI-Nv	14d/2w	3; 42; 600; 1800; 25200	80	iTBS	16	…	98	29	22.1	3.3	3.4	3.2	60.44	14.13	✓	✓	✓
						Inactive Sham	16	…	97	28	26.1	9.7	3.7	4.1	58.75	9.98			
Zhao et al. (2014) China	Sch (DSM-IV); Neg; b-PANSS-N ≥20; …	Circular; …	20d/4w	4; 80; 600; 2400; 48000	80	iTBS	24	0.54	…	…	47.7	11.8	…	…	76.10	8.60	✗	✗	✗
					80	Sham at 180°	22	0.55	…	…	46.7	13.1	…	…	78.30	7.60			
Zhen et al. (2015) China	Sch (DSM-IV);…; IPD	Figure-8; …	20d/4w	2; 40; 600; 1200; 24000	80	iTBS	28	0.39	…	…	41.4	11.2	7.7	2.0	…	…	…	…	…
						Sham at 180°	29	0.48	…	…	45.4	13.5	7.3	2.4	…	…			
Zheng et al. (2012) China	Sch (CCMD-3); …; IPD	Circular; …	5d/4w	2; 10; 600; 1200; 6000	80	iTBS	18	1.00	…	…	56.4	9.3	32.9	8.1	65.20	12.60	✗	✗	✗
						Sham at 180°	17	1.00	…	…	55.6	5.8	31.7	7.2	66.90	12.10			
Brain target: Left inferior frontal gyrus																			
Walther et al. (2020) Switzerland^d^	Sch (DSM-5); …; OPD/IPD	Figure-8; F5/F7/FC5/FT7 Figure-8; CP4/6	1d/1w	1; 1; 600; 600; 600	80	iTBS	20	0.60	479	638	34.3	12.6	11.6	8.9	96.60	24.20	…	…	…
					80	Inactive Sham	20												
Brain target: Cerebellar vermis																			
Basavaraju et al. (2021) India	Sch (DSM-5); Neg; b-SANS each item ≥3; IPD/OPD	Figure-8; MRI-Nv	5d/6w	2; 10; 600; 1200; 6000	100	iTBS	30	0.80	…	…	31.2	9.9	8.4	5.6	…	…	✗	✗	✗
						Inactive Sham	30	0.73	…	…	34.2	8.1	10.9	8.0	…	…			
Chauhan et al. (2021) India	Sch (ICD-10); Pos; TRS; b-BPRS ≥45; …	Figure-8; 1 cm^b^	5d/3w	2; 10; 600; 1200; 6000	80	iTBS	19	0.37	605	122	41.7	8.9	16.1	5.5	93.21	9.41	✗	✗	✗
						Inactive Sham	16	0.47	557	120	39.4	8.2	13.0	7.0	90.59	8.09			
Zhu et al. (2019) China	Sch (ICD-10); …; IPD	Figure-8; 1 cm^b^	10d/2w	1; 10; 600; 600; 6000	100	iTBS	14	0.43	…	…	31.5	4.9	13.9	6.1	…	…	…	…	…
						Sham at 180°	17	0.41	…	…	35.8	3.6	16.0	5.1	…	…			
Zhu et al. (2021) China	Sch (ICD-10); Neg b-PANSS P2 <4; IPD	Figure-8; 1 cm^b^	10d/6m	1; 10; 600; 600; 6000	100	iTBS	32	0.56	464	233	35.2	7.1	15.4	7.8	64.66	1959	✗	✗	✓
						Sham at 180°	32	0.44	487	289	35.3	6.1	15.8	6.5	66.06	19.26			

Abbreviations: Sch, schizophrenia; Pos, prominent positive symptoms; Neg, prominent negative symptoms; TRS, treatment-refractory schizophrenia; CCMD-3, Chinese classification of mental disorders version 3; b-SANS, baseline scale for the assessment of negative symptoms; b-BPRS, baseline brief psychiatric rating scale; b-PANSS, baseline positive and negative syndrome scale; b-PANSS-P, baseline positive subscale of PANSS; b-PANSS-N, baseline negative subscale of PANSS; b-ΔPANSS, baseline improvement of positive and negative syndrome scale; IPD, inpatients; OPD, outpatients; MRI-Nv, magnetic resonance imaging-based neuronavigation; iTBS, intermittent theta-burst stimulation; rMT, resting motor threshold; PPS, pulse per session; PPD, pulse per day; CPZEq, chlorpromazine equivalence dose of adjunctive antipsychotics; … indicates data not available; ✓ indicates significant improvement of symptoms; ✗ indicates no significant change.

a 5 cm or 6 cm anterior to the motor hotspot.

b 1 cm below the inion (centered over midline cerebellum).

c Overall study duration; Tx, Intervention duration; End, endpoint duration; d, days; w, weeks; m, months.

d Crossover design with 3 arms: iTBS over the left inferior frontal gyrus, cTBS over right inferior parietal lobe, and placebo over left inferior parietal lobe.

e Efficacy of symptom improvement was proved by individual study; T, PANSS total scores; P, positive symptoms; N, negative symptoms.

Overall, the 524 included patients were randomized to receive iTBS (*n* = 266) or sham control (*n* = 258). The common durations of treatment in these studies were 2 weeks and 4 weeks (*M* = 2.5, *SD* = 1.4, range = 1 day to 4 weeks). The mean age of the patients was 37.2 years (*SD* = 12.6); approximately 59.02% of the patients were men, with a mean illness duration of 12.5 years (*SD* = 11.5). The mean baseline PANSS total score was 74.94 (*SD* = 19.75) in these patients. The baseline characteristics of patients included in these studies did not significantly differ between the iTBS and sham control groups.

### 3.3 Quality of included studies and risk of bias assessment

The overall risk of bias in the individual studies was low, except for two studies with a high risk of bias because of the high attrition bias (six participants who left the study early were not included in the final analysis and no information was provided regarding which groups those participants were from) (Zhu et al., 2019) and other bias (possible carryover effect in a crossover study design without an adequate washout phase) (Walther et al., 2020). Risks of bias for all included studies are shown in Supplementary Table S1. The quality of evidence according to the GRADE criteria is summarized in Supplementary Table S2. Evidence for the primary and secondary outcomes were of high quality.

### 3.4 Therapeutic efficacy

#### 3.4.1 All studies

The effect of iTBS as an adjunct to antipsychotics on total psychopathology in patients with schizophrenia was significant relative to the sham control (trials = 8, *n* = 315, SMD = −0.92, 95% CI [−1.54, −0.30], *p* = 0.004, *I* ^*2*^ = 84%). The improvement in negative symptoms (trials = 10, *n* = 397, SMD = −1.30, 95% CI [−2.03, −0.56], *p* < 0.001, *I* ^*2*^ = 90%) and general psychopathology (trials = 7, *n* = 305, SMD = −0.58, 95% CI [−1.15, -0.01], *p* = 0.04, *I* ^*2*^ = 82%) was also noticed among patients with schizophrenia who received iTBS. Adjunctive iTBS did not have a marked effect on positive symptoms in patients with schizophrenia; no significant differences in the pooled scores for the positive subscale of PANSS were noted between the iTBS and sham control groups (trials = 9, *n* = 375, SMD = 0.08, 95% CI [–0.35, 0.51], *p* = 0.73, *I* ^*2*^ = 75%).

Several studies have examined the effects of iTBS on cognitive function in patients with schizophrenia. To avoid underpowered results, the pooled results were only considered when at least two adequately powered studies were available (Turner et al., 2013). The results of the pooled analysis of cognitive parameters (verbal fluency test, forward digit span, backward digit span, trail making test A, trail making test B, Stroop inference test, and visuospatial working memory assessment) are summarized in Supplementary Table S3. None of the cognitive parameters were superior in iTBS group to the sham control group.

#### 3.4.2 Left dorsolateral prefrontal cortex

**FIGURE 2:**
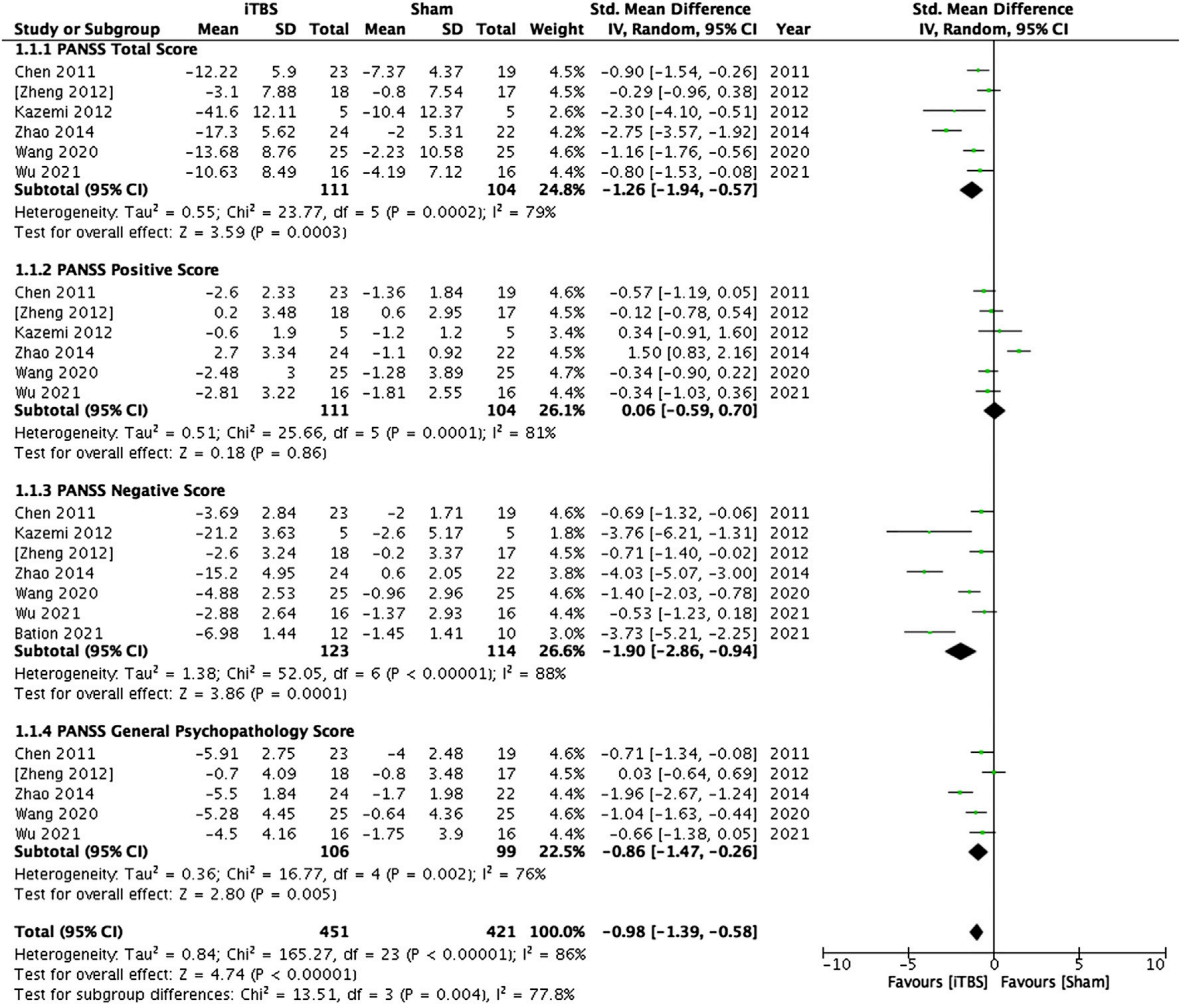
Standardized mean differences for changes in the psychopathology of patients with schizophrenia who received intermittent theta-burst stimulation on the left dorsolateral prefrontal cortex.

Majority of the included studies (*n* = 8) selected left dorsolateral prefrontal cortex (DLPFC) as iTBS stimulation target. Concordance with the results in pooled analysis of all studies, iTBS targeting the left DLPFC had beneficial effects on the total PANSS scores (*p* < 0.001), negative subscale scores (*p* < 0.001), and the general psychopathology subscale score (*p* = 0.005), but not the positive subscale scores (*p* = 0.86) in patients with schizophrenia. Figure 2 presents the results of the pooled analysis of the iTBS studies targeting DLPFC.

#### 3.4.3 Cerebellar vermis

**FIGURE 3:**
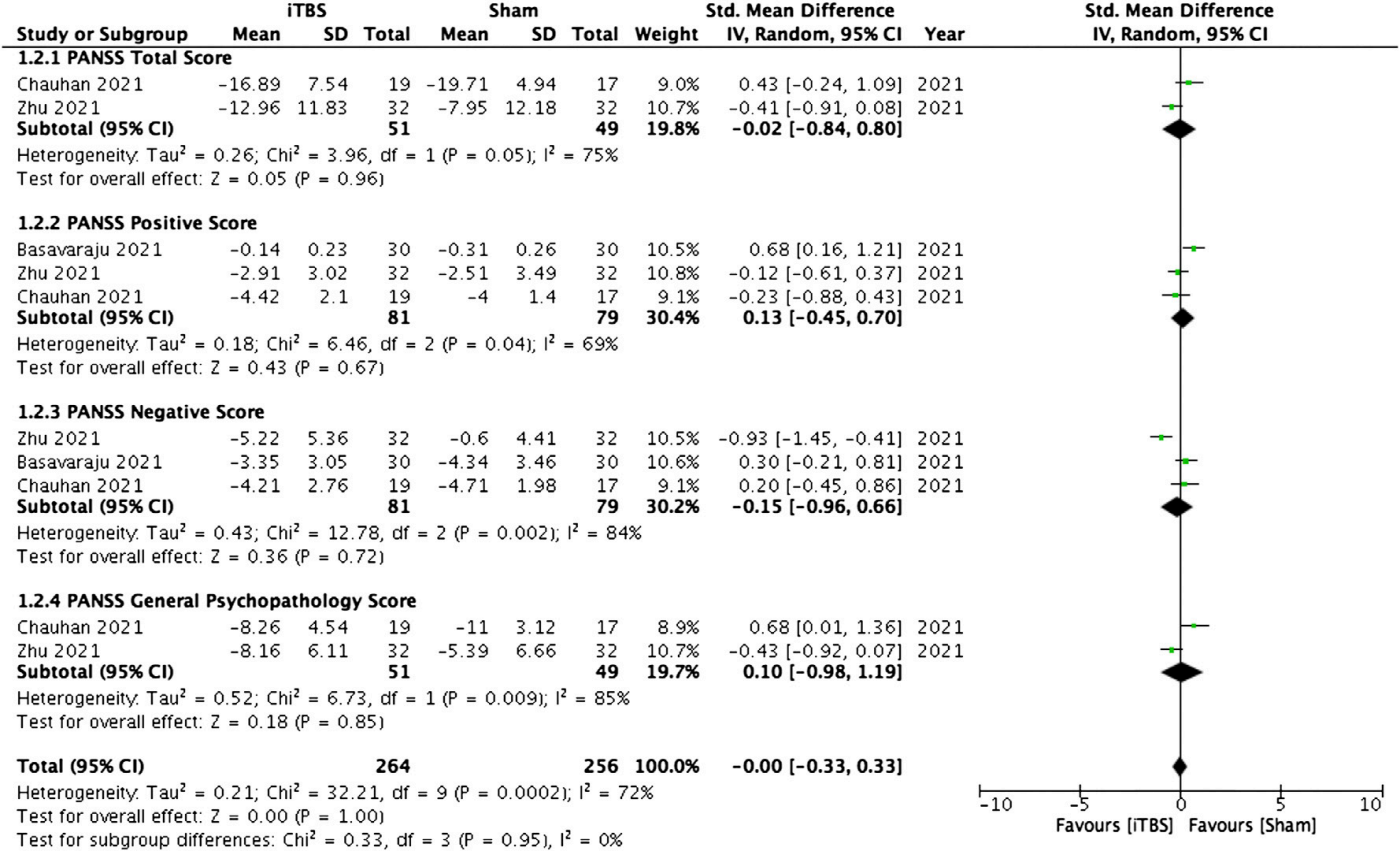
Standardized mean differences for changes in the psychopathology of patients with schizophrenia who received intermittent theta-burst stimulation on the cerebellar vermis.

Four studies examined the effectiveness of iTBS on the cerebellar vermis in patients with schizophrenia. Pooled analysis of these studies showed that cerebellar vermal iTBS failed to yield a significant improvement in all domains of psychopathology measured by PANSS scores (see Figure 3).

#### 3.4.4 Meta-regression analysis

Moderators defined *a priori* were tested in an exploratory meta-regression analysis to examine their effects on the primary and secondary outcomes. No interaction of age, sex, illness duration, overall study duration, or adjunct antipsychotic dosage with the primary or secondary outcomes was observed in this study. In other words, the dose of received antipsychotics has no effect on the effectiveness of iTBS augmentation in patients with schizophrenia. With regard to their baseline psychopathology, we found that the patients’ baseline negative symptoms and general psychopathology had significant effects on the treatment response, in terms of reduction of PANSS total, negative, and general psychopathology scores. This implies that the more severe baseline negative symptoms and general psychopathology of the patients, the more improvement in PANSS total, negative, and general psychopathology scores they are experienced after iTBS augmentation. The stimulation parameters have significantly interacted with the primary outcomes, including total PANSS scores, negative subscale, and general psychopathology subscale, but not with the secondary outcome (discontinuation rate). The results of meta-regression were summarized in Supplementary Table S4. The effects of the stimulation parameters on treatment outcomes are discussed in the following subgroup analyses.

#### 3.4.5 Subgroup analyses of therapeutic efficacy for various stimulation parameters

**FIGURE 4:**
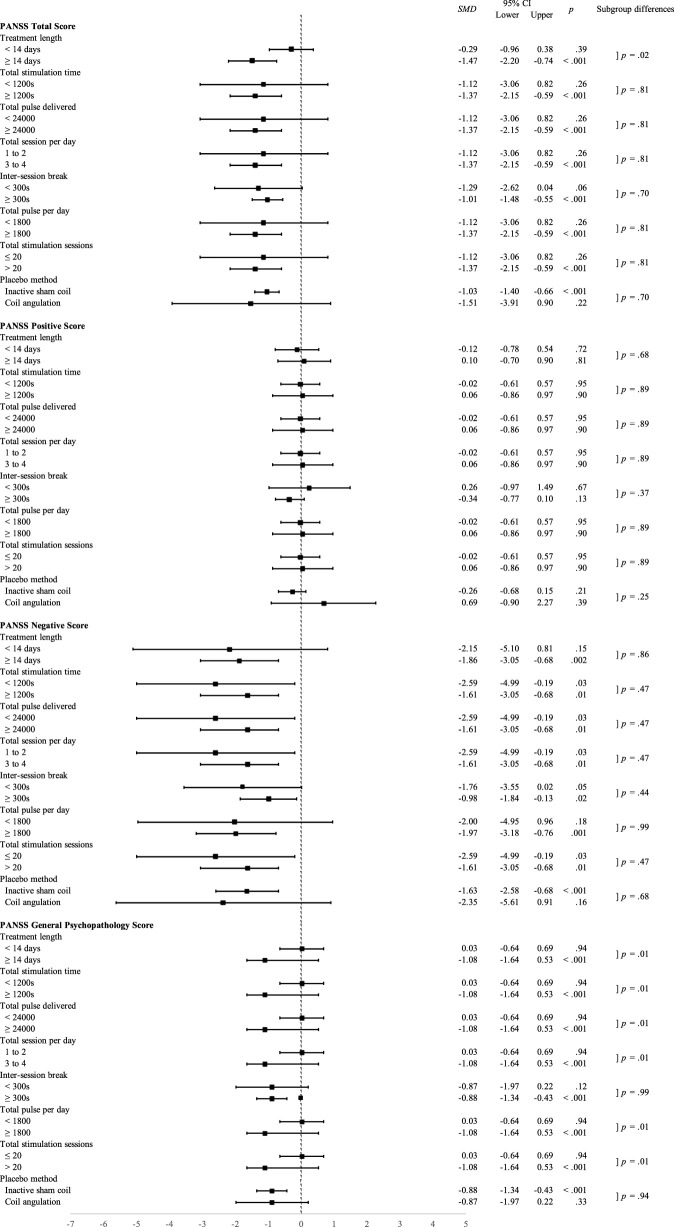
Standardized mean differences for subgroup analyses of therapeutic efficacy of different stimulation parameters of intermittent theta-burst stimulation on the dorsolateral prefrontal cortex.

Stratified meta-analyses of therapeutic efficacy were performed for various stimulation parameters, including targeted brain area, stimulation session, number of stimulation pulses per session, stimulation strength, and type of sham control. Aiming to examine the ideal iTBS paradigm, all studies were categorized according to their brain target. Only studies on DLPFC fulfilled the criteria for subgroup analysis as the studies on cerebellar vermis were too small in studies number. Despite the trends of superiority that could be noticed in several parameters, the subgroup analyses of the parameters mentioned above that showed statistically significant differences between the two groups mainly focused on the general psychopathology. The results are summarized in Figure 4.

##### 3.4.5.1 Stimulation duration

The iTBS was delivered to patients with schizophrenia in 1, 5, 10, 14, and 20 days in different studies. As stratified by the median days, those who received iTBS for more than 14 days showed significantly better results for total PANSS scores (*p* < 0.001), negative symptoms (*p* = 0.002), and general psychopathology (*p* < 0.001) compared with those who received fewer than 14 days treatment, although the subgroup differences were only significance for total PANSS score and general psychopathology.

For the length of stimulation, a longer stimulation period (≥1200 s) was more beneficial in total scores (*p* < 0.001), negative scores (*p* = 0.01), and general scores (*p* < 0.001) of PANSS for patients with schizophrenia under iTBS treatment. Furthermore, those who received more than one session a day, a break for at least 300s produced a more prominent treatment effect on scores of totals (*p* < 0.001), negative (*p* = 0.01), and general (*p* < 0.001) scales of PANSS for patients with schizophrenia. As illustrated in Figure 4, the subgroup difference between different lengths of stimulation was significant for the reduction of general psychopathology.

##### 3.4.5.2 Stimulation sessions

The total number of stimulation sessions delivered to patients with schizophrenia varied from 10, 20, 42, and 80. Patients who received more than 20 sessions of iTBS showed significantly better results for the total PANSS scores (*p* < 0.001), negative symptoms (*p* = 0.01), and general psychopathology (*p* < 0.001) compared with those who received 20 sessions or less. Those patients who received 3 to 4 sessions iTBS a day showed better outcome than those who received 1 to 2 sessions in the total (*p* < 0.001), negative (*p* = 0.01), and general (*p* < 0.001) scales of PANSS. Like the analyses for other stimulation parameters, the significant differences between the two groups of the different numbers of total sessions and the different number of daily sessions were found in the improvement of general psychopathology.

##### 3.4.5.3 Stimulation pulses

Dose-dependent effects of iTBS were indirectly demonstrated in the stratified meta-analysis based on the number of stimulation pulses delivered per day. Patients with schizophrenia who received more than 1800 pulses in a day exhibited greater improvements in total PANSS scores (*p* < 0.001), negative symptoms (*p* = 0.001), and general psychopathology (*p* < 0.001) compared with those who received fewer than 1800 pulses per session. Also, those patients who received a total number of more than 24000 pulses showed more prominent improvement in total PANSS scores (*p* < 0.001), negative symptoms (*p* = 0.001), and general psychopathology (*p* < 0.001) as compared with those who received fewer pulses. The between-groups difference in dose-dependent effects of iTBS was statistically significant in the reduction of general psychopathology.

##### 3.4.5.4 Stimulation strength

Among all included studies, most of the studies applied the stimulation strength at 80% of the resting motor threshold (rMT) whilst only three studies delivered stimulation at 100% rMT. In the subgroup analysis of stimulation strength, classified according to the targeted brain area, all studies that targeted DLPFC delivered 80% rMT stimulation strength. To avoid underpowered results, there was an inadequate number of studies to perform subgroup analysis.

##### 3.4.5.5 Type of sham control

Both inactive sham coil and coil angled away from the scalp were used in the included studies as sham controls. The use of an inactive sham coil resulted in a more robust effect on the reduction of total PANSS scores (*p* < 0.001), negative subscale scores (*p* < 0.001), and general subscale (*p* < 0.001) compared with the application of an angled coil, although none of the subgroup differences between these two methods were significant.

### 3.5 Safety profile

#### 3.5.1 Discontinuation

No differences were observed between the iTBS and sham control groups in terms of all-cause discontinuation of treatment [trials = 12, *n* = 494, RR = 0.80, 95% CI (0.46, 1.37), *p* = 0.42, *I* ^*2*^ = 0%] or discontinuation due to adverse events (trials = 9, *n* = 370, RR = 0.36, 95% CI [0.08, 1.59], *p* = 0.18, *I* ^2^ = 0%). In total, one patient from the iTBS group (1 headache) and four patients from the sham control group (1 headache, 3 psychosis exacerbation) in the included studies withdrew because of adverse events.

#### 3.5.2 Adverse events

Overall, iTBS was well tolerated in patients with schizophrenia. No significant difference in the reported adverse events was observed between the iTBS and sham control groups, although the incidences of headache, pain, dizziness, and nausea were slightly marked. Two patients with schizophrenia emergent hypomania/mania symptoms while receiving iTBS of cerebellar vermis were worth noticing (Basavaraju et al., 2020). The reported adverse events are summarized in Table 2.

**TABLE 2:** Adverse events reported in patients with schizophrenia treated with intermittent theta-burst stimulation.

	Trials	Intervention			Placebo			RR (95%CI)	*p*
		Events	Total	%	Events	Total	%		
Dizziness	1	3	32	9.38	0	32	0.00	7.00 [0.38, 130.26]	0.19
Fatigue	1	5	30	16.67	4	20	20.0	1.25 [0.39, 3.99]	0.71
Headache	4	9	86	10.47	5	78	6.41	1.61 [0.59, 4.37]	0.35
Mania	1	2	30	6.67	0	30	0.00	5.00 [0.25, 99.95]	0.29
Nausea	2	7	52	13.46	1	52	1.92	5.00 [0.92, 27.24]	0.06
Pain	2	6	52	11.54	2	52	3.85	2.20 [0.51, 9.42]	0.29
Psychosis	2	0	35	0.00	3	29	10.34	0.21 [0.02, 1.82]	0.16

### 3.6 Sensitivity analysis

Multiple sensitivity analysis was conducted using an *a priori* protocol to examine the heterogeneity of the included studies. Overall, most of the outcomes measured in this study remained unchanged, and none of the significant effects of iTBS on total PANSS scores and negative symptoms in patients with schizophrenia disappeared. The results are summarized in Supplementary Table S5. The benefit of the iTBS on the general psychopathology in patients with schizophrenia was diminished after excluding studies with a high risk of bias and studies that designated non-psychopathology as the primary outcome, therefore the results of general psychopathology should be interpreted cautiously with the consideration of the unobserved heterogeneity in those included studies.

### 3.7 Publication bias

Publication bias was assessed both qualitatively (through funnel-plot asymmetry) and quantitatively (through Begg and Mazumdar rank correlation, Egger’s regression, the Fail-safe N test, and the trim and fill method). Details of the publication bias analysis are summarized in Supplementary Table S6. Results of Duval and Tweedie’s trim and fill method revealed no evidence of publication bias for the primary or secondary outcomes.

## 4 Discussion

This meta-analysis evaluated recent RCTs on the efficacy and safety of iTBS augmentation in patients with schizophrenia. iTBS adjunct to antipsychotics was associated with significant improvements in the psychopathology in patients with schizophrenia, particularly for negative symptoms and general psychopathology, but not for positive symptoms or cognitive function. This result was consistent with the studies that revealed the efficacy of rTMS for alleviating negative symptoms in patients with schizophrenia (Osoegawa et al., 2018; Tseng et al., 2022). In addition, our findings are in line with a meta-analysis on the efficacy of TBS for the treatment of patients with depression (Chu et al., 2021); the close relationships between depressive symptoms and negative symptoms in schizophrenia sometimes make them hardly distinguishable (Krynicki et al., 2018). More specifically, delivering iTBS with 1800 pulses over left DLPFC is proven to be effective in the treatment of depression (Li et al., 2018; Li C. T et al., 2020). Shared pathophysiology in brain circuits, for example, DLPFC, between schizophrenia and depression may suggest the same iTBS paradigm work for both patient populations (Hui et al., 2021; Rolls, 2021). Furthermore, in the evaluation using PANSS, items of general psychopathology are comprised of and overlapped with several depressive symptoms, for example, depression, guilt feelings, poor attention, and active social avoidance. In the present study, the improvement in depressive symptoms in patients with schizophrenia who received iTBS augmentation was represented by a reduction in the PANSS negative subscale score and general psychopathology. It is believed that these improvements might be a combination of the direct effects on negative symptoms and antidepressant effects.

The present results showed that iTBS augmentation did not improve positive symptoms in patients with schizophrenia. Consistent with our findings, a meta-analysis of 11 RCTs reported that rTMS improved auditory hallucinations in patients with schizophrenia; however, this result was not stable after a sensitivity analysis (Li J et al., 2020). iTBS delivered to the DLPFC and cerebellar vermis may not be the effective brain targets, as other brain regions, for example, the temporoparietal cortex, should be considered. Therefore, additional studies of iTBS efficacy in alleviating positive symptoms in schizophrenia are warranted.

Consistent with the result of a meta-analysis of rTMS effect on cognitive function in patients with schizophrenia (Sciortino et al., 2021), we found that adjunctive iTBS did not improve cognitive function. Nevertheless, due to the limited evidence and relatively small number of studies examining cognitive function, it cannot exclude that specific dimensions of cognitive functions may respond differently to iTBS.

The optimal protocol of iTBS for schizophrenia (e.g., targeted brain area, stimulation duration, stimulation sessions, and stimulation pulses) remains to be determined. Preliminary, our results may provide a shred of evidence to find the ideal iTBS paradigm. Our results revealed that iTBS targeted on the left DLPFC could ameliorate the psychopathology in patients with schizophrenia. Dysfunctional activations of the ventrolateral prefrontal cortex and medial prefrontal cortex are associated with negative and positive symptoms, respectively, whereas abnormalities in the DLPFC have been associated with disorganized symptoms and social perception (Goghari et al., 2010; Shin et al., 2015). Normalization of excitability in the frontal cortex is one of the main positive effects of schizophrenia treatments, including antipsychotics and noninvasive brain stimulations (Kani et al., 2017). More specifically, increased resting functional connectivity between the left DLPFC and brain regions that encompass dopamine neuron cell bodies after stimulation are indicating significant modulation of dopamine transmission by iTBS (Bation et al., 2021). The restoration of abnormal connectivity of mesocorticolimbic dopamine pathways may partly answer the improvement of negative symptoms and general psychopathology of patients with schizophrenia who received iTBS that was observed in this meta-analysis (McCutcheon et al., 2019; Xu et al., 2019).

Our results revealed that patients who received more than 20 iTBS sessions, at least 1200 s of iTBS, at least 24000 pulses, for at least 14 days exhibited greater improvements in psychopathology. For the intensity of the iTBS, patients who received at least 1800 pulses with 3–4 sessions a day showed better psychopathology outcomes. Similarly, a study reported that iTBS (600 vs. 1800 pulses) dose-dependently enhanced cortical excitability and functional connectivity in the motor cortex of healthy subjects (Nettekoven et al., 2014). However, another study reported that iTBS-induced neuroplasticity was reversed with prolonged stimulation (600 vs. 1200 pulses) (Gamboa et al., 2010). An animal study revealed that iTBS-induced cortical protein expression was not an accumulative dose-dependent effect, but distinct profiles with threshold characteristics and a waxing-and-waning effect were observed (Volz et al., 2013). This effect pattern was observed in the subgroup analysis of negative symptoms of patients with schizophrenia who received iTBS on DLPFC. In contrast to what has been observed in analyses for total PANSS score and general psychopathology, the effect sizes in the improvement of negative symptoms were more robust in those parameters of lower intensity (< 1800 pulses and only 1 to 2 sessions a day) and shorter period (≤20 sessions, <1200s of iTBS, <24000 pulses, <14 days), although the between-groups differences were not statistically significant and patients in both subgroups are all showed a reduction in PANSS negative score. The small number of included studies limited further exploratory analysis but future investigations on the heterogeneity of treatment response is warranted.

Studies using an inactive sham coil reported a significant improvement in the psychopathology compared with those flipped coils to realize the effect of sham stimulation. Broadbent et al. (2011) reported that people randomized to active rTMS were more likely to correctly guess group randomization than those allocated to sham stimulation. People receiving inactive sham coil stimulation may have an inadequate placebo effect due to the lack of sensation on the skull in contrast to active iTBS. In the angled-coil method, substantial cortical stimulation may occur, especially in 45° coil arrangements, with approximately half the potential for inducing motor-evoked potentials over the motor cortex as active rTMS (Lisanby et al., 2001). Thus, the angled-coil method may induce a partially active placebo that could bias the results (Loo et al., 2000). These shortcomings of both sham methods could cause partial blinding success and bias the estimations of treatment efficacy.

In addition, iTBS seems to be safe and well-tolerated for patients with schizophrenia. No differences in terms of discontinuation or adverse events were found between the iTBS and sham control groups.

This meta-analysis had some limitations. First, the uneven sample size and population distribution of the included studies may have affected the validity and generalizability of the outcomes. Furthermore, differences in baseline characteristics, diagnostic tools, stages of illness, and the adjunctive antipsychotics used may have influenced the results. Second, the treatment duration and endpoints varied among the included studies. Knowledge concerning the timepoints at which the after-effect of iTBS occurs and the duration of the effect is lacking. Most of the included studies reported acute treatment effects of iTBS on schizophrenia; thus, the optimal maintenance duration of iTBS remains unclear. Therefore, long-term iTBS studies on schizophrenia are required in the future. Third, all the studies included iTBS adjunct to antipsychotics for treating patients with schizophrenia. This limited the interpretation of the efficacy of iTBS as a monotherapy.

## Conclusion

Based on the evidence obtained in this meta-analysis, iTBS adjunct to antipsychotic treatment is associated with a significant improvement in negative symptoms in patients with schizophrenia and has favorable tolerability. iTBS targeting the left DLPFC with more than 1800 pulses per day and more than 20 sessions might be the optimal protocol. The results of this meta-analysis may provide insights into the use of iTBS as an additional treatment for schizophrenia for alleviating negative symptoms.

## Data Availability

The original contributions presented in the study are included in the article/Supplementary Material, further inquiries can be directed to the corresponding author.
