# Supplementary material for: Efficacy and safety of intermittent theta-burst stimulation in patients with schizophrenia: A meta-analysis of randomized sham-controlled trials
Source: Front Pharmacol. 2022 Aug 22;13:944437. doi: 10.3389/fphar.2022.944437 (PMC9441632; doi:10.3389/fphar.2022.944437)
Supplement: Supplementary file 1 [file DataSheet1.zip › Supplement 1.DOCX]

**Supplement 1.** Risk of bias summary of all included studies.
