# Supplementary material for: Efficacy and safety of intermittent theta-burst stimulation in patients with schizophrenia: A meta-analysis of randomized sham-controlled trials
Source: Front Pharmacol. 2022 Aug 22;13:944437. doi: 10.3389/fphar.2022.944437 (PMC9441632; doi:10.3389/fphar.2022.944437)
Supplement: Supplementary file 1 [file DataSheet1.zip › Supplement 2.DOCX]

**Supplement 2**. Summary of findings of GRADE appraisal criteria.

| Outcomes | Anticipated absolute effects* (95% CI) | | Relative effect (95% CI) | № of participants  (studies) | Quality of evidence (GRADE) |
| --- | --- | --- | --- | --- | --- |
|  | Placebo | Risk difference with iTBS |  |  |  |
| PANSS total scores | - | SMD -0.92  (-1.54 to -0.30) | - | 315 (8 RCTs) | ⨁⨁⨁⨁ HIGH |
| *Left dorsolateral prefrontal cortex* |  | SMD -1.26  (-1.94 to -0.57) |  | 215 (6 RCTs) | ⨁⨁⨁⨁ HIGH |
| *Cerebellar Vermis* |  | SMD -0.02  (-0.84 to 0.80) |  | 100 (2 RCTs) | ⨁⨁⨁⨁ HIGH |
| PANSS positive scale | - | SMD 0.08  (-0.35 to 0.51) | - | 375 (9 RCTs) | ⨁⨁⨁⨁ HIGH |
| *Left dorsolateral prefrontal cortex* |  | SMD 0.06  (-0.59 to -0.70) |  | 215 (6 RCTs) | ⨁⨁⨁⨁ HIGH |
| *Cerebellar Vermis* |  | SMD 0.13  (-0.45 to 0.70) |  | 160 (3 RCTs) | ⨁⨁⨁⨁ HIGH |
| PANSS negative scale | - | SMD -1.30  (-2.03 to -0.56) | - | 397 (10 RCTs) | ⨁⨁⨁⨁ HIGH |
| *Left dorsolateral prefrontal cortex* |  | SMD -1.90  (-2.86 to -0.94) |  | 237 (7 RCTs) | ⨁⨁⨁⨁ HIGH |
| *Cerebellar Vermis* |  | SMD -0.15  (-0.96 to 0.66) |  | 160 (3 RCTs) | ⨁⨁⨁⨁ HIGH |
| PANSS general psychopathology | - | SMD -0.58  (-1.15 to -0.01) | - | 305 (7 RCTs) | ⨁⨁⨁⨁ HIGH |
| *Left dorsolateral prefrontal cortex* |  | SMD -0.86  (-1.47 to -0.26) |  | 205 (5 RCTs) | ⨁⨁⨁⨁ HIGH |
| *Cerebellar Vermis* |  | SMD 0.10  (-0.98 to 1.19) |  | 100 (2 RCTs) | ⨁⨁⨁⨁ HIGH |
| All-cause discontinuation | 116 per 1,000 | 93 per 1,000  (53 to 159) | RR 0.80  (0.46 to 1.37) | 494 (12 RCTs) | ⨁⨁⨁⨁ HIGH |
| *Left dorsolateral prefrontal cortex* | 104 per 1,000 | 49 per 1,000  (22 to 110) | RR 0.47  (0.21 to 1.05) | 334 (9 RCTs) | ⨁⨁⨁⨁ HIGH |
| *Cerebellar Vermis* | 139 per 1,000 | 173 per 1,000  (82 to 358) | RR 1.24  (0.59 to 2.57) | 160 (3 RCTs) | ⨁⨁⨁⨁ HIGH |
| Discontinuation due to adverse event | 28 per 1,000 | 10 per 1,000  (2 to 44) | RR 0.36  (0.08 to 1.59) | 370 (9 RCTs) | ⨁⨁⨁⨁ HIGH |
| *Left dorsolateral prefrontal cortex* | 408 per 1,000 | 15 per 1,000  (3 to 78) | RR 0.37  (0.07 to 1.98) | 210 (6 RCTs) | ⨁⨁⨁⨁ HIGH |
| *Cerebellar Vermis* | 13 per 1,000 | 4 per 1,000  (0 to 100) | RR 0.33  (0.01 to 7.89) | 160 (3 RCTs) | ⨁⨁⨁⨁ HIGH |
| *The risk in the intervention group (and its 95% confidence interval) is based on the assumed risk in the comparison group and the relative effect of the intervention (and its 95% CI). CI = Confidence interval; SMD = Standardized mean difference; RR = Risk ratio | | | | | |
