# Supplementary material for: Efficacy and safety of intermittent theta-burst stimulation in patients with schizophrenia: A meta-analysis of randomized sham-controlled trials
Source: Front Pharmacol. 2022 Aug 22;13:944437. doi: 10.3389/fphar.2022.944437 (PMC9441632; doi:10.3389/fphar.2022.944437)
Supplement: Supplementary file 1 [file DataSheet1.zip › Supplement 4.DOCX]

**Supplement 4**. Regression analysis for effect of intermittent theta-burst stimulation on psychopathology and discontinuation of patients with schizophrenia.

|  | Total PANSS score | | PANSS positive score | | PANSS negative score | | PANSS general score | | All-cause treatment discontinuation | | Discontinuation due to adverse event | |
| --- | --- | --- | --- | --- | --- | --- | --- | --- | --- | --- | --- | --- |
| Parameters | Coefficient | *p* | Coefficient | *p* | Coefficient | *p* | Coefficient | *p* | Coefficient | *p* | Coefficient | *p* |
| Age | 0.075 | .11 | -0.002 | .99 | 0.086 | .06 | 0.081 | .10 | -0.013 | .84 | -0.028 | .24 |
| Male | -3.053 | .46 | -1.357 | .44 | -1.012 | .82 | -3.060 | .48 | -2.648 | .30 | -1.563 | .21 |
| Baseline PANSS total | 0.003 | .51 | -0.002 | .89 | 0.019 | .14 | 0.001 | .81 | -0.002 | .97 | -0.014 | .25 |
| Baseline PANSS positive | 0.017 | .62 | 0.014 | .60 | 0.009 | .83 | 0.026 | .39 | 0.002 | .98 | -0.011 | .39 |
| Baseline PANSS negative | 0.067 | .006 | -0.063 | .16 | 0.103 | .05 | 0.035 | .04 | -0.053 | .43 | -0.028 | .38 |
| Baseline PANSS general | 0.118 | .005 | -0.049 | .26 | 0.136 | .016 | 0.108 | .002 | 0.046 | .51 | -0.024 | .41 |
| Duration of illness | 0.079 | .28 | 0.007 | .83 | 0.083 | .25 | 0.079 | .33 | 0.006 | .95 | -0.068 | .24 |
| Antipsychotics dose | 0.001 | .49 | 0.001 | .87 | -0.001 | .23 | 0.002 | .78 | 0.004 | .47 | -0.002 | .30 |
| Overall study duration | -0.052 | .20 | -0.020 | .35 | -0.026 | .59 | -0.052 | .25 | -0.058 | .42 | -0.037 | .40 |
| Stimulation parameters |  |  |  |  |  |  |  |  |  |  |  |  |
| % rMT | 0.660 | .36 | 0.266 | .63 | 1.094 | .23 | 0.187 | .84 | 1.105 | .09 | -0.060 | .97 |
| Total sessions per day | -0.367 | .11 | 0.070 | .76 | -0.297 | .42 | -0.482 | .05 | -0.516 | .16 | 0.285 | .64 |
| Inter-session breaks | 0.001 | .10 | 0.001 | .43 | 0.001 | .16 | 0.001 | .12 | 0.001 | .09 | NA | |
| Pulse per day | -0.001 | .11 | 0.001 | .76 | -0.001 | .42 | -0.001 | .05 | -0.001 | .09 | 0.001 | .85 |
| Treatment lengths | -0.132 | < .001 | 0.016 | .69 | -0.160 | .002 | -0.112 | .00 | -0.055 | .34 | 0.093 | .56 |
| Total stimulation sessions | 0.001 | .79 | 0.001 | .38 | 0.001 | .09 | 0.001 | .98 | 0.001 | .11 | 0.001 | .55 |
| Total stimulation time | -0.022 | .007 | 0.004 | .65 | -0.024 | .05 | -0.020 | .01 | -0.019 | .17 | 0.013 | .59 |
| Total pulses delivered | -0.001 | .007 | 0.001 | .65 | -0.001 | .05 | -0.001 | .01 | -0.001 | .13 | 0.001 | .64 |
