# Supplementary material for: Efficacy and safety of intermittent theta-burst stimulation in patients with schizophrenia: A meta-analysis of randomized sham-controlled trials
Source: Front Pharmacol. 2022 Aug 22;13:944437. doi: 10.3389/fphar.2022.944437 (PMC9441632; doi:10.3389/fphar.2022.944437)
Supplement: Supplementary file 1 [file DataSheet1.zip › Supplement 5.DOCX]

**Supplement 5.** Sensitivity analysis of study outcomes.

|  | **Total PANSS scores** | **PANSS positive subscale scores** | **PANSS negative subscale scores** | **PANSS general subscale scores** | **All-cause Discontinuation** | **Discontinuation due to adverse events** |
| --- | --- | --- | --- | --- | --- | --- |
| SMD / RR | -0.92 [-1.54, -0.30]^***^ | 0.08 [-0.35, 0.51] | -1.30 [-2.03, -0.56]^***^ | -0.58 [-1.15, -0.01]^*^ | 0.80 [0.46, 1.37] | 0.36 [0.08, 1.59] |
| MD / OR | -7.75 [-12.93, -2.57]^**^ | 0.05 [-0.82, 0.91] | -4.88 [-7.75, -2.02]^***^ | -1.92 [-3.68, -0.17]^*^ | 0.75 [0.40, 1.41] | 0.34 [0.07, 1.60] |
| Alternative statistical approach  (fixed effects models) | -0.75 [-0.99, -0.51]^***^ | 0.07 [-0.14, 0.28] | -0.77 [-0.99, -0.55]^***^ | -0.57 [-0.80, -0.33]^***^ | 0.75 [0.45, 1.26] | 0.34 [0.08, 1.42] |
| Study quality  (excluding high risk of bias studies) | -0.95 [-1.66, -0.23]^**^ | 0.13 [-0.34, 0.60] | -1.16 [-1.89, -0.42]^***^ | -0.57 [-1.23, 0.09] | 0.85 [0.47, 1.54] | 0.36 [0.08, 1.59] |
| Study design  (excluding crossover study) | … | … | … | … | 0.80 [0.46, 1.37] | 0.36 [0.08, 1.59] |
| Study population  (excluding study designated non-psychopathology as primary outcome) | -1.08 [-1.91, -0.24]^*^ | 0.17 [-0.37, 0.70] | -1.68 [-2.83, -0.52]^**^ | -0.68 [-1.44, 0.07] | 0.80 [0.45, 1.43] | 0.36 [0.08, 1.59] |
| Study size  (excluding small sample size studies) | -0.82 [-1.45, -0.18]^*^ | 0.06 [-0.40, 0.51] | -1.16 [-1.89, -0.42]^**^ | … | 0.80 [0.46, 1.37] | 0.36 [0.08, 1.59] |
| Publication language  (excluding Chinese language publication) | -1.02 [-1.73, -0.32]^**^ | 0.10 [-0.38, 0.59] | -1.39 [-2.21, -0.56]^**^ | -0.68 [-1.31, -0.05]^*^ | 0.80 [0.45, 1.43] | 0.36 [0.08, 1.59] |
| Diagnostic criteria  (excluding diagnostic criteria other than DSM) | -1.47 [-2.20, -0.74]^***^ | 0.20 [-0.46, 0.87] | -1.78 [-2.89, -0.67]^**^ | -1.08 [-1.64, -0.53]^***^ | 0.82 [0.44, 1.53] | 0.37 [0.07, 1.98] |
| ^*^*p* < .05; ^**^*p* <.01; ^***^*p* < .001; SMD: Standardized mean differences; MD: Mean differences; RR: Risk ratio; OR: Odds ratio | | | | | | |
