# Supplementary material for: Efficacy and safety of intermittent theta-burst stimulation in patients with schizophrenia: A meta-analysis of randomized sham-controlled trials
Source: Front Pharmacol. 2022 Aug 22;13:944437. doi: 10.3389/fphar.2022.944437 (PMC9441632; doi:10.3389/fphar.2022.944437)
Supplement: Supplementary file 1 [file DataSheet1.zip › Supplement 6.DOCX]

| Outcomes | Begg and Mazumdar rank correlation  (Kendall's 𝜏) | Egger’s regression  (Intercept) | Fail-safe N  (N to bring ⍺ > 0.05) | Trim and Fill method  (Observed value, 95%CI)  (Adjusted value, 95%CI) |
| --- | --- | --- | --- | --- |
| PANSS total score | -0.39 | -4.48 | 105 | -0.89; -1.46 to -0.32  -0.89; -1.46 to -0.32 |
| PANSS positive | 0.11 | 0.47 | 0 | 0.08; -0.36 to 0.52  0.08; -0.36 to 0.52 |
| PANSS negative | -0.33 | -6.70 | 137 | -1.13; -1.87 to -0.40  -1.37; -2.21 to -0.53 |
| PANSS general | 0.05 | -1.67 | 36 | -0.59; -1.17 to -0.01  -0.59; -1.17 to -0.01 |
| All-cause treatment discontinuation | -0.44 | -0.20^*^ | 7 | 0.74; 0.40 to 1.40  0.74; 0.40 to 1.40 |
| Discontinuation due to adverse event | -0.33 | -5.63 | 0 | 0.34; 0.07 to 1.60  0.34; 0.07 to 1.60 |
| **p* < .05 | | | | |

**Supplement 6.** Publication bias analysis.
