# Supplementary figures and images for: Efficacy and safety of intermittent theta-burst stimulation in patients with schizophrenia: A meta-analysis of randomized sham-controlled trials
Source: Front Pharmacol. 2022 Aug 22;13:944437. doi: 10.3389/fphar.2022.944437 (PMC9441632; doi:10.3389/fphar.2022.944437)

**Supplement 3.** Standardized mean difference for changes in cognitive function.


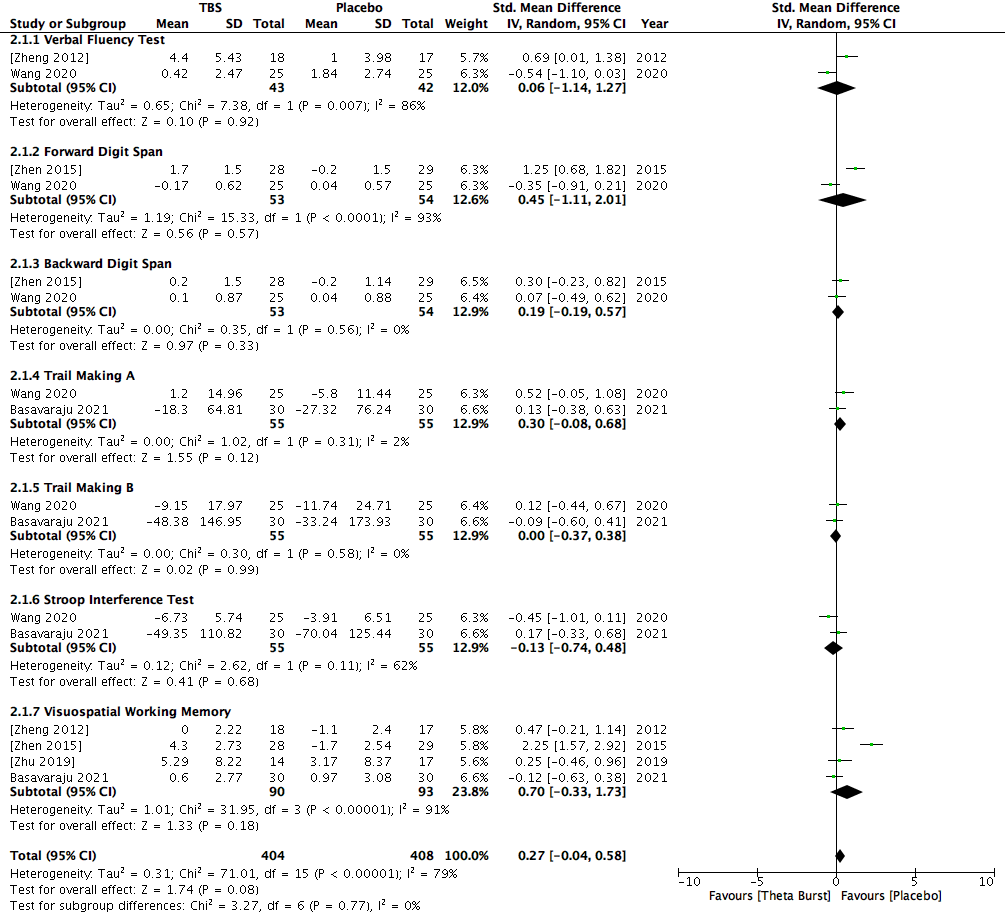

Supplement: Supplementary file 1 [file DataSheet1.zip › Supplement 3.DOCX]
